# Circulating protein and antibody biomarker for personalized cancer immunotherapy

**DOI:** 10.1186/s40425-016-0150-0

**Published:** 2016-08-16

**Authors:** Jianda Yuan

**Affiliations:** Oncology Clinical Research, Merck Research Laboratories, Rahway, 07065 NJ USA

**Keywords:** Antibody response, Antigen discovery, Mass spectrometry, Liquid biopsy, Biomarker and personalized cancer immunotherapy

## Abstract

Immune checkpoint blockade therapies are revolutionizing standard cancer treatments. Immune checkpoint inhibitors likely function to enhance the tumor specific antigen response in order to achieve favorable clinical outcomes. Thus, continuous efforts to identify the common tumor-specific antigens are essential for the broad clinical application of these therapies. Several immunoproteomics approaches have been used in order to screen for this specificity. In a recent article from Jhaveri and colleagues published in the February issue of *Cancer Immunology Research*, antibody biomarkers were screened in pancreatic cancer patients who received allogeneic, granulocyte-macrophage colony stimulating factor-secreting pancreatic cancer vaccine (GVAX) by using a serum antibody-based SILAC immunoprecipitation (SASI) approach. Using this assay, several new tumor antigens (MYPT1, PSMC5 and TRFR) were identified that were found to have significantly different expression in tumors compared with normal tissue. Moreover, patients with detectable antibodies showed improved disease-free survival after GVAX therapy. These targets need to be further validated to determine the full spectrum of tumor antigen immunogencity and their potential clinical application. In addition to antibodies, circulating protein, DNA and RNA in peripheral blood are under clinical investigation as liquid biopsies and have the potential to provide guidance for future personalized cancer immunotherapy.

## Background

Immune checkpoint blockade therapies are revolutionizing standard cancer treatments [1]. It is a paradigm shift within cancer immunotherapy from a focus on stimulating the immune system to a focus on releasing the checkpoint inhibitors resulting in adequate immune and tumor responses [2]. Tumor rejection antigens allow tumors that are distinct from normal tissue to activate the immune system and generate robust anti-tumor responses [3]. Immune checkpoint blockade enhances the tumor rejection response mediated by these antigens to achieve favorable clinical outcomes. Therefore, it is of importance to identify the right tumor rejection antigens to provide important therapeutic guidance for future cancer immunotherapy. Novel high-throughput technology such as whole exome sequencing allows the systemic analysis of the mutation load of the tumor as well as the identification of potentially immunogenic neoantigens [4]. However, the current data illustrate that the majority of mutated antigens are not shared between patients, and are instead patient specific [5]. Thus, continuous efforts to identify common tumor-specific antigens are essential for the broad clinical application of these therapies.

It is a long standing interest in the potential of antibodies to search for tumor specificity. As Dr. Lloyd J Old highlighted in his G.H.A Clowes Memorial lecture three decades ago, “Antibodies might reveal something specific, something unique about the cancer cell…The antigenic determinants were recognized by antibody” [6]. Several immunoproteomics approaches including *Ser*ologic *P*roteome *A*nalysis (SERPA), *Ser*ological analysis of *re*combinant cDNA *ex*pression libraries (SEREX) and protein microarrays have been explored extensively to find tumor associated antigens and their cognate antibodies [7]. SERPA is a robust immunoproteomics assay to screen antibody reactivity profiles in sera from patients with various diseases. SEREX is another approach to assess the antibody profile in patient sera by using proteins employed on a membrane. NY-ESO-1 was the first cancer testis antigen discovered by SEREX technology. The application of SERPA and SEREX technologies is relatively limited due to the assay specificity and the complexity of the assay preparation and procedure. In addition, the proteins on SEREX membrane expressed by tumor cDNA phase library in bacterial do not account for human posttranslational modification.

Protein microarrays are based upon the development of DNA microarray techniques and have thousands of purified proteins immobilized on a solid surface. Protein microarrays are generally classified into three types: analytical, functional and reverse-phase protein microarrays. ProtoArray®, a functional array, offers a unique approach to analyze the serological response against thousands of protein at the same time. This approach is limited by the number of proteins coated on the slides (e.g., not all human proteins are present, and mutated or modified versions of proteins may not be available) [7].

## Main text

In the February issue of *Cancer Immunology Research* [8], Jhaveri and colleagues used quantitative seroproteomics to identify antibody biomarkers in pancreatic cancer patients treated with allogeneic, granulocyte-macrophage colony stimulating factor-secreting pancreatic cancer vaccine (GVAX). The authors took advantage of stable, isotope-labeled amino acids (SILAC) in pancreatic ductal adenocarcinoma cell culture, immune precipitation with patient-derived antibodies and mass spectrometric analysis. They developed a serum antibodies-based SILAC immuneprecipitation (SASI) approach to identify antibody response elicited by the vaccination.

In this study, pre-vaccine sera was intentionally subtracted from post-vaccine sera in order to assess the vaccine-induced specific antibody responses. In doing so, a few antibodies were identified as targets from post-vaccination samples in patients with favorable clinical outcome. The expression of three antigens (MYPT1, PSMC5 and TRFR) was measured in tumor and normal duct epithelium, and significant differences were found in the expression of these three antigens in tumor compared with normal tissue. Moreover, patients with detectable identified antibodies showed improved disease-free survival.

Overall, the SASI approach was found to identify new tumor antigens as potential biomarkers and therapeutic targets. This approach could also be applied to other similar clinical studies without protein synthesis, but these new targets require further validation as possible pancreatic cancer biomarkers. The caveat and potential limitation of this study is the subtraction of pre-vaccine sera. It limits the ability to identify the baseline antibody response, which may predict the patient’s response to GVAX vaccination. In addition, allogeneic tumor cells instead of autologous tumor cell lines were used for the vaccination. Thus, targets from autologous tumor cells may be partially missed because of the limitation of the allogeneic tumor immunogenicity profile.

## Perspective and future directions

The SASI approach is an effective method to identify tumor-specific antigens, especially common tumor rejection antigens that would allow for the development of “off-the-shelf” vaccinations. In addition to the validation of the expression and distribution of these new targets, it is of importance to further characterize these antibodies and the antigen-specific CD4+ and CD8+ T cell response. The dissociation between antibody responses and antigen-specific CD8^+^ T-cell responses is frequently observed with other tumor antigens. CTLA-4 blockade induced a broad antibody response in cancer patients with ovarian, prostate cancer and melanoma [9, 10]. Advanced melanoma patients with integrated immune responses to NY-ESO-1 antigen had a favorable clinical course after ipilimumab treatment [11]. The majority of NY-ESO-1 seropositive patients without detectable NY-ESO-1–specific CD8^+^ T cells did not experience clinical benefit. Therefore, cellular tumor antigen-specific CD4+ and CD8+ T cell response needs to be evaluated to obtain the full spectrum of the identified antigens immunogencity and explore the potential clinical application.

Antibodies are useful for the discovery of tumor-specific antigens. Moreover, antibodies may be able to directly or indirectly eliminate tumor cells through opsonization, antigen presentation to T cells and by initiating NK cells or complement-dependent cell toxicity [12]. Several potential clinical applications of antibodies including antibody-drug conjugates, antibody cytokine fusions and bispecific/multispecific antibodies are under clinical investigation. A low success rate of current monoclonal antibody therapy is likely due to low sensitivity and specificity. Sensitivity and specificity of the target is critical for successful application [13].

In addition to antibodies, proteins circulating in blood could be potential biomarkers for cancer immunotherapy. As an example, patients with low baseline vascular endothelial growth factor (VEGF) experienced better clinical outcome in advanced melanoma patients treated with ipilimumab. Thus, serum VEGF may be a predictive biomarker for ipilimumab treatment [14]. With advances in mass spectrometry-based serum assays, automated database search algorithms and the proteome discoverer software platform, a mass spectrometry-based serum assay was recently developed to predict clinical outcome in patients treated with PD-1 blockade [15]. Fifty-nine mass spectral (MS) selected from 351 MS identified from the results of baseline serum were defined as DBX008+ and DBX008-. Patients with DBX008+ have a better time to tumor progression and overall survival than patients with DBX008-. Similar to VEGF, these MS themselves in the peripheral blood may have immunomodulatory impacts on human immune cells. The amount of these MS may also be associated with immune suppression or activation in tumor microenvironment. Further characterization of these MS will provide additional information to understand mechanism of action in these patients treated with immune checkpoint blockade.

Besides proteins and antibodies, tumor cells can also release DNA and RNA into the blood by a variety of microvesicle-dependent or -independent mechanisms. Novel technologies with minimal specimen requirements allow the characterization of these tumor derived DNA and RNA. Liquid biopsies including cell-free circulating tumor DNA (ctDNA) from plasma have been investigated for non-invasive detection and monitoring of patient tumors as well as potential biomarkers for cancer immunotherapies [16, 17]. The validation of these assays focused on the serial analyses of DNA, RNA and protein/antibody in peripheral blood will allow us to identify changes in the tumor in real time, detect tumor response or relapse early, identify potential pharmacodynamic/pharmacokinetic predictive biomarkers related to the treatment, understand the resistance mechanisms and provide guidance for future monotherapy or combination personalized cancer immunotherapy.
